# Selective Acetylene Hydrogenation: Influence of Carbon Supports on the Stabilization of Pd_4_S-like Active Sites

**DOI:** 10.3390/nano16030157

**Published:** 2026-01-23

**Authors:** Eduardo Campos-Castellanos, Inmaculada Rodríguez-Ramos, Miguel A. Bañares, Antonio Guerrero-Ruiz, María V. Morales

**Affiliations:** 1 Departamento de Química Inorgánica y Química Técnica, Facultad de Ciencias, UNED, Av. De Esparta s/n 28232 las Rozas, 28232 Madrid, Spain; camposeduardo@ccia.uned.es; 2 Instituto de Catálisis y Petroleoquímica, CSIC, C/Marie Curie 2, Cantoblanco, 28049 Madrid, Spain; irodriguez@icp.csic.es (I.R.-R.); miguel.banares@csic.es (M.A.B.)

**Keywords:** acetylene, hydrogenation, carbon support, palladium sulfide

## Abstract

This study examines how both the nature of the carbon support and the palladium precursor influence catalytic performance in acetylene hydrogenation. Six Pd-based catalysts were prepared on four carbon materials—high-heat-treated fibers (HHTs), carbon nanotubes, activated carbon and high surface area graphite—using either sulfate or chloride precursors. Catalytic tests performed in a continuous fixed-bed reactor reveal that HHT-supported catalysts achieve the highest ethylene selectivity and long-term stability, while in general catalysts derived from sulfate precursors exhibit enhanced selectivity compared to their chloride-derived counterparts. These improvements are consistent with the formation of sulfur, which may be incorporated as sub-stoichiometric sulfide species (S^2−^) interacting with metallic Pd, as revealed by the XPS results, rather than to palladium dispersion alone. The role of the carbon support in stabilizing these sites was further assessed by complementary characterization techniques, including transmission electron microscopy, energy-dispersive X-ray spectroscopy, X-ray photoelectron spectroscopy and Raman spectroscopy. The combined results indicate that highly graphitic supports such as HHT fibers favor sulfur retention at the catalyst surface, thereby promoting the stability and catalytic performance of Pd–S active motifs during acetylene hydrogenation.

## 1. Introduction

The catalytic transformation of pure acetylene remains an area of ongoing research, with a notable focus on its selective hydrogenation to ethylene, as well as its dimerization of acetylene to obtain mainly 1-butene and 1,3-butadiene (C4s) [1,2,3,4,5]. Historically, noble metal-based catalysts, specifically palladium (Pd), have predominated in this field at both industrial and academic levels [6,7,8,9,10,11,12]. In some cases, palladium catalysts are intentionally modified by the incorporation of sulfur atoms [13]. This modification, often described as a controlled form of poisoning, can enhance catalyst selectivity by suppressing undesired reaction pathways. In this sense, our group has reported [14] the development of monometallic Pd catalysts supported on high-surface-area graphite for the selective hydrogenation of butadiene. They further demonstrated that the intentional poisoning of Pd catalysts with sulfur components, achieved by contaminating the catalysts with dibenzothiophene, led to the formation of sulfide species that drastically altered the product distribution of the reaction. Adding Cu resulted in less overhydrogenation (less butane), more 1-butene production and less 2-butenes, or at least a higher proportion of 1-butene over these, and better catalyst stability. The addition of sulfur-containing compounds to Pd blocks certain catalytic sites, which reduces butane formation and increases selectivity toward butenes. It also alters the ratio between the different isomers (1-butene, cis/trans 2-butenes), generally favoring partial hydrogenation and possibly a higher yield of 1-butene.

The utilization of carbonaceous materials as a support for metallic nanoparticles is a prevalent strategy in the preparation of heterogeneous catalysts. This widespread adoption is, in part, attributable to the moderate and weak interaction between the metal and the carbonaceous support, which facilitates the feasible synthesis of novel chemical compositions, such as intermetallic compounds or the action of promoters/poisons. Such weak interactions also enable the formation of additional phases, including transition metal sulfides. Furthermore, the functionality of the carbon surface has proven to be an effective method for anchoring metallic nanoparticles onto the carbon substrate [15,16,17,18,19].

In this context, the nature of the Pd precursor and the type of carbon support play a critical role in defining the final catalytic properties. Previous investigations of our research group have demonstrated the formation, from the sulfate precursor and after reduction treatment, of a new active palladium sulfide phase, Pd_4_S, which was highly selective in different partial hydrogenations [14,20,21,22]. In this line of research, recent articles [15,16,23] studied new methods of preparing these palladium sulfide particles on carbonaceous fibers heated at high temperature, which were analyzed in the partial hydrogenation of butadiene, obtaining highly satisfactory results in terms of selectivity toward 1,3-butadiene and butenes. Also, our study [23] demonstrated/identified the formation of the specific Pd_4_S phase for the first time. A.J. McCue et al. [21] implemented a series of monometallic catalysts prepared with commercially available bulk phase metal sulfide powders (PdS, CuS and Ni_2_S_3_) and tested them in different partial hydrogenation reactions of alkynes to alkenes, achieving very promising results and bringing more visibility to the usually ignored metal sulfides. Recently, another external group has also demonstrated the possibility of the formation of these highly selective palladium sulfide particles from other different precursors, as is the case with PdCl_2_(PhS-C_6_H_4_-4-NH_2_) [24].

Applications of those catalysts formed by the active phase Pd_4_S supported on carbon nanofibers have been reported [22], for instance in the selective hydrogenation of alkynes to alkenes, leading in some cases to high selectivities of products such as propylene, if methylacetylene, propadiene, propylene and propane are included in the reactive mixture. In the same line, Y. Liu et al. [25] compared Pd-based catalysts with Pd_4_S-based materials in the partial hydrogenation of external and internal butynes. They reported that conventional Pd catalysts typically reach alkene selectivities of only 25–35%, whereas Pd_4_S-based catalysts achieve selectivities exceeding 90%.

Recently, X. Zhao et al. [11] conducted a review of advances related to Pd-based catalysts used in selective hydrogenation reactions, where they discuss how poisoning Pd catalysts with sulfur is an important innovative advance in improving selectivity toward certain products, forming a highly selective active phase such as Pd_4_S. D. Albani et al. [26] developed a straightforward post-treatment in which palladium nanoparticles supported on graphitic carbon nitride are exposed to aqueous sodium sulfide, directing the formation of a nanostructured Pd_3_S phase. These catalysts were subsequently applied to alkyne semi-hydrogenations. In this study, they compared the Pd_3_S and Pd_4_S phases with that of lead-modified and monometallic Pd catalysts supported on different materials, concluding that the most promising results are achieved with catalysts containing the Pd_3_S and Pd_4_S phases.

More recently, W. Jing et al. [27] demonstrated a compelling approach by surface-modifying Pd_4_S nanocrystals with PPh_3_ ligands, resulting in a catalyst with excellent catalytic activity and durable selectivity for the semi-hydrogenation of terminal alkynes. Z. Wu et al. [28] developed Pd_4_S catalysts to carry out selective hydrogenation of nitroarenes, with high selectivity of more than 98% at complete conversion under mild conditions. V. H. Nissinen et al. [29] revealed important aspects of the regeneration process of sulfur-poisoned Pd-rich methane combustion catalysts. The results obtained in this study show the way to a more comprehensive understanding of the regeneration of Pd-based catalysts. Continuing with the regeneration processes, previous articles have developed a top-down strategy for the simultaneous degradation of Pd sulfides and creation of palladium single-atom sites. Through a one-step thermal atomization process, Pd_4_S model nanoparticles were successfully converted into single-atom palladium sites supported on nitrogen-and-sulfur-co-doped carbon (Pd_1_/N, S-C) after being loaded onto a ZIF-8 framework and subjected to thermal treatment [30,31].

Pd sulfides are also used beyond partial hydrogenation, such as the hydrogen evolution reaction (HER). For example, X. Liu et al. [32] developed palladium nickel sulfide catalysts with excellent results in terms of the activity of HER. Obviously, the Pd precursor can influence the metal’s particle size, its oxidation state, and its interaction with the support during synthesis, which in turn affects Pd dispersion and the availability of active sites. Similarly, the intrinsic properties of supports based on carbon materials, such as specific surface area, porosity, surface functionalization, and electrical conductivity, can modulate the nucleation, growth, and stabilization of Pd nanoparticles. A thorough understanding of how these factors influence catalytic selectivity and activity is therefore fundamental for optimizing Pd catalyst design.

The main objective of this work is to elucidate, for the first time in acetylene hydrogenation, how the nature of the carbon support governs the stabilization of sulfur-modified Pd surface ensembles and, consequently, their impact on catalytic selectivity and stability. To this end, the catalysts were systematically studied using a combination of advanced characterization techniques, including transmission electron microscopy (TEM), energy-dispersive X-ray spectroscopy (EDX), X-ray photoelectron spectroscopy (XPS) and Raman spectroscopy. In particular, this study aims to evaluate how the nature of the carbon support influences the chemical environment, distribution and stability of these Pd–S surface ensembles, which have previously been associated with high catalytic activity and selectivity in acetylene hydrogenation.

## 2. Materials and Methods

### 2.1. Catalyst Synthesis

#### 2.1.1. Support

In this work, four different carbon supports have been used: high surface area graphite (G), carbon nanofibers (CNFs), carbon nanotubes (CNTs) and activated carbon (AC). The G (S_BET_ = 396 m^2^/g) exhibits a highly ordered structure and non-porous surface morphology and was supplied by TIMREX S.A. (Bodio, Switzerland). The commercial CNFs, specifically the Pyrograph III PR24-HHT (S_BET_ = 32 m^2^/g), were provided by Applied Sciences Inc. According to the supplier, the PR24-HHT fibers (referred to as HHTs) underwent a high-temperature treatment (approximately 3000 °C). These fibers are characterized by a stacked-cup morphology, featuring a hollow core that runs along their entire length. Additionally, their outer surface is jagged and exhibits ‘round head’ or ‘loop’ structures that serve to connect multiple layers [33]. Multiwall CNTs (3100 Series, 95% purity), obtained from Nanocyl (Sambreville, Belgium) (S_BET_ = 285 m^2^/g) and hereafter named as CNTs, possess multiple walls with a hollow inner core and both of their ends closed. Finally, the high-purity AC (S_BET_ = 1190 m^2^/g), derived from olive stones and supplied by Oleícola El Tejar (Córdoba, Spain), was pretreated with hydrochloric acid in order to remove residual inorganic impurities.

#### 2.1.2. Synthesis of Pd Catalysts

Pd catalysts were prepared with a 1 wt% palladium loading.

For the PdCl_2_ precursor, it was first dissolved in concentrated HCl to form dihydrogentetrachloropalladate (H_2_[PdCl_4_]). The solution was then heated to remove chlorine, dissolved in distilled water, dried, and subsequently diluted in water to achieve the final desired volume. This resulting solution was then added dropwise onto the carbon nanofibers, or the carbon nanotubes, by incipient wetness impregnation. After this preparation, two catalysts were obtained: PdCl/HHT and PdCl/CNT.

When PdSO_4_ was used as the precursor, the catalysts were prepared via incipient wetness impregnation using aqueous solutions. After impregnation, the samples were dried at 100 °C in an oven for 24 h. Before starting with catalytic tests and characterization, the catalysts were reduced in a glass reactor under hydrogen for 2 h at 300 °C (5 °C/min). Following this preparation, four catalysts were obtained: PdS/HHT, PdS/CNT, PdS/AC and PdS/G. It should be clarified that the notation “PdS” refers to the sulfate precursor (PdSO_4_) rather than the stoichiometry of the resulting catalytic phase.

### 2.2. Catalyst Characterization

Various characterization techniques were employed to investigate the presence and stabilization of sulfur-modified palladium species consistent with Pd_4_S-like active sites.

Metallic particle size was determined using a JEOL JEM-2100 transmission electron microscope (JEOL Ltd., Tokyo, Japan) equipped with a field-emission gun and operated at 200 kV. The particle size distributions were obtained by measuring the diameters of Pd nanoparticles directly from the TEM micrographs using the ImageJ software 1.54G. In addition to the TEM images, the chemical composition of samples was also analyzed by EDX.

XPS was also utilized to ascertain the Pd/S surface ratios and chemical states. Spectra were acquired with a SPECS GmbH ultra-high vacuum (UHV) system, equipped with a PHOIBOS 150 9MCD energy analyzer (SPECS Group, Berlin, Germany) and a non-monochromatic Mg X-ray source (200 W, 12 kV). Prior to XPS analysis, the reduced catalysts were pressed into 10 mm diameter pellets and outgassed for 20 h to achieve a dynamic vacuum below 10^−10^ mbar. The resulting spectra were analyzed using Casa XPS software 2.3.25PR1.0, with binding energies corrected to the C 1s peak at 284.6 eV.

The samples separated after being used in the reaction were also analyzed using Raman spectroscopy. The equipment was a Renishaw Qontor Raman system (Renishaw, Wotton-under-Edge, UK). The excitation line was 514 nm solid-state laser, with 2 mW on the sample, a 20× objective, and an acquisition time of 10 s. Spectra were analyzed with Renishaw WiRE and open-source Orange Data Mining software 3.38.1. 

### 2.3. Catalytic Evaluation of the Gas-Phase Partial Hydrogenation of Acetylene

Before starting the reaction, any mass-transfer limitation was hampered using the adequate catalyst grain size (sieve fraction 0.35–0.5 mm). The prepared catalyst was pretreated and reduced inside the reactor itself under H_2_ atmosphere (50 cm^3^/min of a mixture of hydrogen/acetylene/nitrogen in a proportion of 4:1:45) at 300 °C for 2 h. The acetylene hydrogenation reaction was performed in a continuous flow fixed-bed tubular reactor placed in a PID Eng&Tech system, equipped with a furnace with highly precise control of temperatures and mass flow controllers. Catalytic tests were caried out feeding the reactor with H_2_:C_2_H_2_ in a 4:1 molar ratio using a total gas flow of 50 cm^3^/min. Catalytic tests started when the catalyst reached the required reaction temperature (40 °C). Conversion measurements were performed every 20 min. Acetylene conversions were determined through real-time analysis of the exhaust gas using a Varian CP-3800 gas chromatograph (Varian Inc., Walnut Creek, CA, USA) equipped with a Flame Ion Detector (FID). A GS-alumina capillary column (aluminum oxide with proprietary deactivation), characterized by the highest retention of olefins relative to comparable paraffins, with dimensions of 50 m × 0.535 mm × 10 μm, was employed. Acetylene conversion, selectivity and activity were calculated as follows:

Conv C_2_H_2_ (%) = (([C_2_H_2_]^o^ − [C_2_H_2_]^f^)/[C_2_H_2_]^o^) × 100 (1)

Conv C_2_H_2_ (%) (1) is the acetylene conversion percentage, and [C_2_H_2_]^o^ and [C_2_H_2_]^f^ are referred to as the incoming and outgoing C_2_H_2_ concentrations, respectively.

Selectivity C_2_H_6_ (%) = (([C_2_H_6_])/∑_i_ [products]_i_) × 100 (2)

Selectivity C_2_H_6_ (%) in Equation (2) is the percentage of ethane-produced [C_2_H_6_], referred to as the concentration ∑_i_ [products]_i_ including ethane, ethylene and C4 products. The concentration of ethane produced and ∑_i_ [products]_i_ is the total concentration of the products (ethane, ethylene and C4_s_).

Selectivity C_2_H_4_ (%) = (([C_2_H_4_])/∑_i_ [products]_i_) × 100 (3)

Selectivity C_2_H_4_ (%) in Equation (3) is the percentage of ethylene-produced [C_2_H_4_], referred to as the concentration ∑_i_ [products]_i_ including ethane, ethylene and C4 products.

Selectivity C4s (%) = ((∑_i_ [C4_s_] × 2)/∑_i_ [products]_i_) × 100 (4)

Selectivity C4s (%) (4) is the percentage of all the C4s produced, mainly butadiene and 1-butene [C4s], referred to as the concentration of C4s produced, and ∑_i_ [products]_i_ is the total concentration of the products.

Activity (μmol C_2_H_2_ converted/g_catalyst_ ∙ s) = (([C_2_H_2_]^o^ − [C_2_H_2_]^f^ × 1000)/g_catalyst_ × 60) (5)

Activity (μmol C_2_H_2_ converted/g_catalyst_ ∙ s) (5) is the catalytic activity; [C_2_H_2_]^o^ and [C_2_H_2_]^f^ refer to the incoming and outgoing C_2_H_2_ concentrations, and g_catalyst_ is the mass of the catalyst.

C. B. (%) = ((∑_i_ [products]_i_)/([C_2_H_2_]^o^)) × 100 (6)

C. B. (%) (6) is the carbon mass balance, ∑_i_ [products]_i_ is the total concentration of the products and [C_2_H_2_]^o^ refers to the incoming C_2_H_2_ concentrations.

## 3. Results and Discussion

### 3.1. Catalytic Results

A comprehensive summary of the activities, selectivity values toward ethane, ethylene and C_4_s, and carbon balance of the different catalysts studied is provided in Table 1. Carbon balances remain above 90% for all experiments. Regarding catalytic activity, the highest values are obtained for catalysts prepared from the sulfate precursor when supported on G and AC.

**Figure 1:**
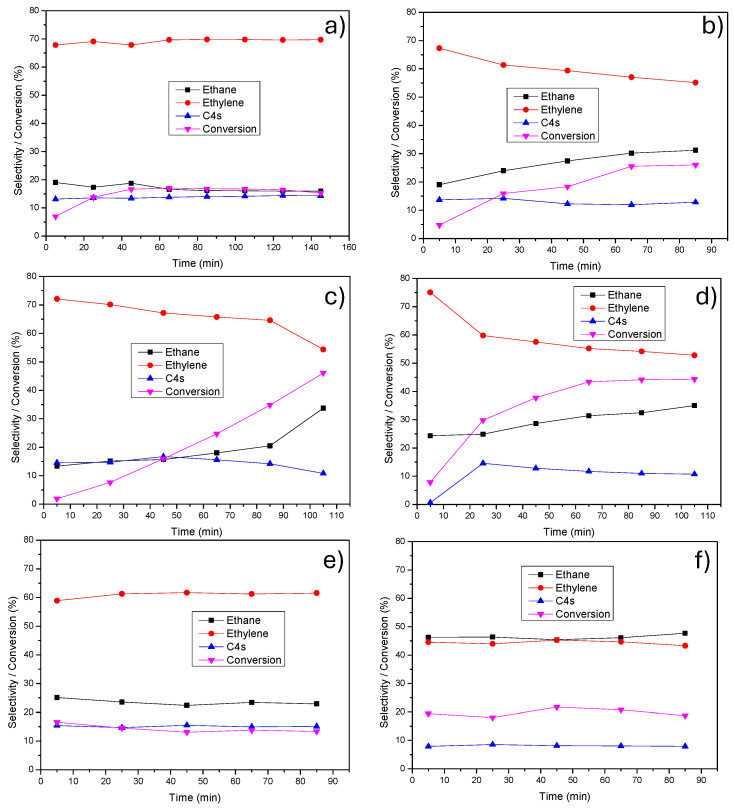
Time-on-stream evolution of ethane, ethylene and C_4_ selectivities and acetylene conversion at 40 °C for: (**a**) PdS/HHT, (**b**) PdS/CNT, (**c**) PdS/AC, (**d**) PdS/G, (**e**) PdCl/HHT and (**f**) PdCl/CNT.

When sulfate- and chloride-derived catalysts are compared on the same support, the presence of sulfur generally results in lower ethane formation and higher ethylene selectivity, indicating partial suppression of overhydrogenation. This effect is particularly evident for the CNT-supported systems. However, the data also show that initial selectivity values alone do not fully describe catalyst performance, as pronounced differences in selectivity stability are observed. An important outcome of Table 1 is that only the sulfate-derived catalyst supported on HHT fibers (PdS/HHT) maintains constant ethylene and ethane selectivities with time-on-stream, which is consistent with the stabilization of sulfur-modified Pd surface ensembles previously reported by our research group [15,21,23]. In contrast, sulfate-derived catalysts supported on CNT, AC and G exhibit a progressive increase in ethane selectivity accompanied by a decrease in ethylene selectivity, as indicated by the ranges reported and discussed in Figure 1. This demonstrates that the beneficial effect of sulfur strongly depends on its stabilization by the carbon support. In contrast, chloride-derived catalysts, particularly PdCl/CNT, show substantially higher ethane selectivity, indicating enhanced total hydrogenation in the absence of sulfur-modified Pd sites.

Concerning C_4_ selectivity, the highest values are obtained for HHT-supported catalysts (PdS/HHT and PdCl/HHT), suggesting that the low surface area and highly graphitized nature of this support favors dimerization pathways through the intrinsic properties of the active Pd sites rather than diffusion limitations. Since C_4_ products appear to be mainly associated with secondary or parallel surface-mediated reactions following ethylene formation, their selectivity is closely linked to the yield of unsaturated C_2_ species and the stabilization of surface reaction intermediates at the relatively low reaction temperature (40 °C).

Figure 1 presents the stability results for the catalysts prepared from the sulfate precursor, detailing their conversion and selectivity toward ethylene, ethane, and C4 products over time on stream. The most significant finding is that the catalyst supported on HHT fibers, despite yielding a lower overall conversion, demonstrates remarkable stability in its ethylene (70%) and ethane selectivity (14%) over time. Conversely, the other three ex-sulfate catalysts with supports of CNT, AC and G exhibit a clear trend of decreasing ethylene selectivity and increasing ethane selectivity over the same period. The formation of C_4_ products—mainly 1-butene and 1,3-butadiene, with smaller amounts of cis-2-butene and trans-2-butene—remained relatively constant throughout the reaction. While these selectivity results have been compared at relatively low conversion levels, previous studies confirm that the catalyst prepared from the sulfate precursor and supported on HHT fibers maintains its stable selectivity, even at higher conversion rates [10]. A recent study [34] investigated phenylene-sulfide modifiers of different chain lengths—oligomers (OPS) and polymers (PPS)—to tune the surface of Pd catalysts. Three systems (Pd/Al_2_O_3_, Pd@OPS-2/Al_2_O_3_ and Pd@PPS), all with ~5 nm Pd nanoparticles, were tested at 100 °C for acetylene hydrogenation. Although all maintained near-complete conversion, ethylene selectivity declined over time. The best catalyst, Pd@PPS, reached a final ethylene selectivity of 68%. In comparison, our PdS/HHT catalyst shows lower ethane formation (14% vs. their ~20%), indicating reduced overhydrogenation, and displays a similar C_4_ selectivity (16% vs. their 15%).

A previous study on palladium catalysts supported on activated carbon [35] investigated the evolution of different Pd sulfide phases as a function of thermal treatment and their impact on activity, selectivity and stability in acetylene partial hydrogenation. The authors reported that PdS remains stable in the absence of thermal treatment or after reduction in H_2_ below 150 °C, whereas treatment at 200–250 °C leads to the formation of intermediate sulfide phases (Pd_16_S_7_ with contributions from Pd_4_S). Reduction at higher temperatures (300–350 °C) promotes the transformation toward Pd_4_S-rich materials. Since the catalysts in the present work were reduced at 300 °C in H_2_, these results are consistent with the possible formation of sulfur-modified Pd phases enriched in Pd_4_S-like motifs. Importantly, the same study demonstrated a clear correlation between sulfur content, sulfide phase evolution and catalytic performance: sulfur-rich PdS phases exhibited low conversion and ethylene selectivities of around 60%, and intermediate sulfur contents led to higher conversions with ethylene selectivities of approximately 75%, whereas sulfur-poor Pd sulfide phases achieved near-complete conversion with ethylene selectivities of 80–90%. In addition, W. Zhang et al. [36] developed a series of Pd/WO_3_ and Pd/WS_2_ catalysts with different Pd loadings, identifying the 0.8 Pd/WS_2_ catalyst as the most efficient. This material displayed high stability at full conversion and maximum ethylene selectivities of up to 70%, values that are comparable to, but do not exceed, those obtained with the PdS/HHT catalyst in the present study. Notably, these reactions were carried out at room temperature, i.e., under conditions very close to those employed here (40 °C).

The different evolutions of selectivity will be discussed in greater depth when the characterization results are presented (see below).

### 3.2. Catalyst Characterization

#### 3.2.1. TEM-EDX

**Figure 2:**
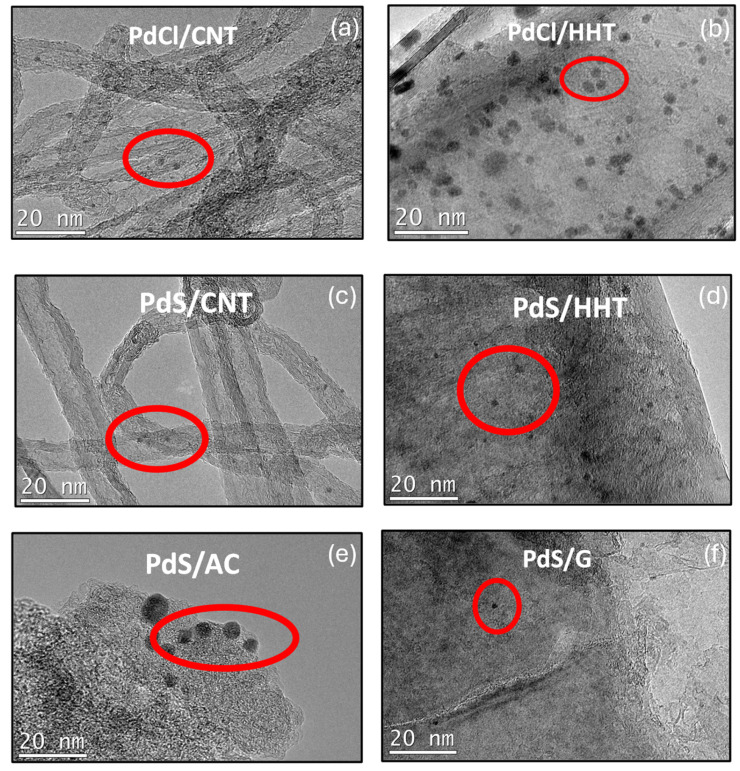
TEM images of the post-reaction samples.

**Figure 3:**
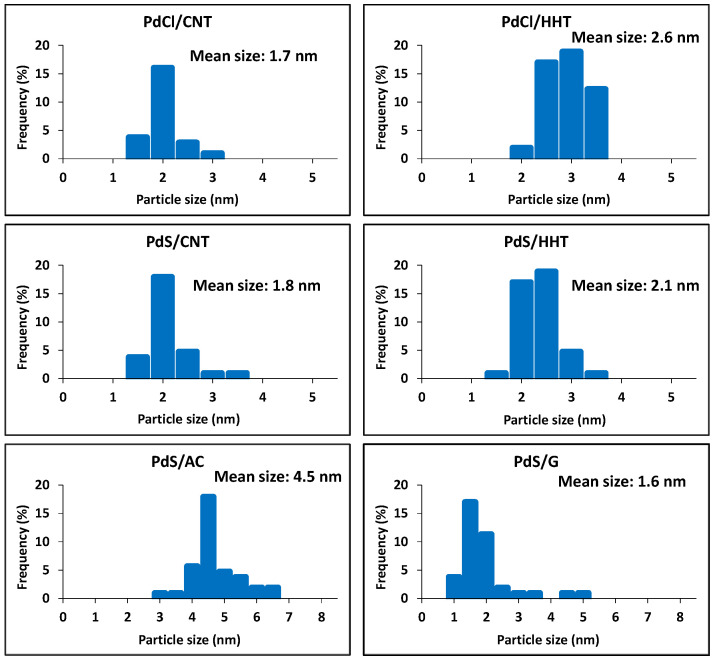
Histograms of the particle sizes distribution of the catalysts measured by TEM.

An analysis of the structural properties of the catalysts after reaction was carried out by TEM (Figure 2). The images clearly show that, in all cases, the palladium nanoparticles are uniformly dispersed over the different carbonaceous supports. Particle size distributions obtained from these micrographs are summarized in the histograms of Figure 3. With the exception of the PdS/AC sample, all catalysts present a narrow and homogeneous distribution, with average particle sizes in the range of 1.6–2.6 nm. In contrast, the PdS/AC catalyst exhibits significantly larger nanoparticles, with a mean diameter of 4.5 nm, more than twice the size observed for the other systems. These results highlight the strong influence of the support on nanoparticle stabilization, with activated carbon being less effective in preventing particle growth under the reaction conditions.

This larger Pd particle size observed in the catalyst supported on activated carbon is attributed to the greater structural and chemical heterogeneity of this support. The irregular distribution of the precursor in the porous network and the presence of surface oxygenated groups lead to less controlled nucleation and more extensive nanoparticle growth. In contrast, more graphitic supports (graphite, carbon nanotubes and HHT fibers) favor a more uniform dispersion of the precursor and more homogeneous nucleation, resulting in smaller and more uniformly sized particles [37,38,39].

Post-reaction samples were further characterized by TEM–EDX mapping to confirm the presence of sulfur originating from the sulfate precursor. As shown in the EDX spectra (Figure S1), the catalyst supported on HHT fibers exhibited the highest sulfur content among the three catalysts prepared from the sulfate precursor. This sample was also the most selective, supporting prior research that highlights the high selectivity of palladium sulfide nanoparticles. Conversely, the catalysts supported on CNT, AC and G showed a low or nearly nonexistent presence of sulfur, which may account for their initial selectivity followed by a shift toward total hydrogenation, resulting in a greater yield of ethane over time.

One possible explanation for the higher sulfur content in HHT fibers compared with CNTs and AC is that HHT is an extremely graphitized carbon material. Its surface consists almost exclusively of ordered graphitic planes, with very few defects or surface oxygenated groups [40]. Consequently, the ionic anchoring of the PdSO_4_ precursor is limited due to the absence of functional groups (e.g., carboxyls or hydroxyls), and electron transfer is also hindered because HHT does not expose graphite edge sites. As a result, part of the sulfate anion may not fully decompose or be removed during reduction and can be retained on the HTT carbon surface, as evidenced by XPS (see below). This leads to a longer retention of the sulfate species, which can then be partially transformed into Pd–S phases during reduction treatment or under reaction conditions [41]. By contrast, AC contains abundant oxygenated groups and has a larger surface area (mainly composed of micropores) that promote the ionic adsorption of Pd^2+^ and sulfate, while also facilitating the complete reduction of Pd and the removal of sulfur residues during thermal treatment. Therefore, the amount of residual S is low. Although CNTs are also graphitic, they have more structural defects (edges, vacant sites and curvatures) than HHT fibers. These defects act as anchoring sites for the precursor, helping to achieve more efficient reduction and more complete decomposition of the sulfate, thereby reducing the residual S content. In contrast, HHT fibers, being the most orderly support with the lowest defect density and lowest surface area, do not offer these active sites for sulfate decomposition, retaining more sulfur after use in the reaction or after reduction treatment [42,43].

EDX spectra of the catalysts prepared from the chloride precursor (Figure S1) showed a complete absence of chlorine, while the palladium signal was more intense than in the sulfate-derived catalysts. This observation correlates well with their catalytic performance: the higher palladium availability, together with the absence of sulfur acting as a poison, led to a decrease in ethylene selectivity, preferentially promoting total hydrogenation and resulting in higher ethane yields.

#### 3.2.2. Surface Properties

**Figure 4:**
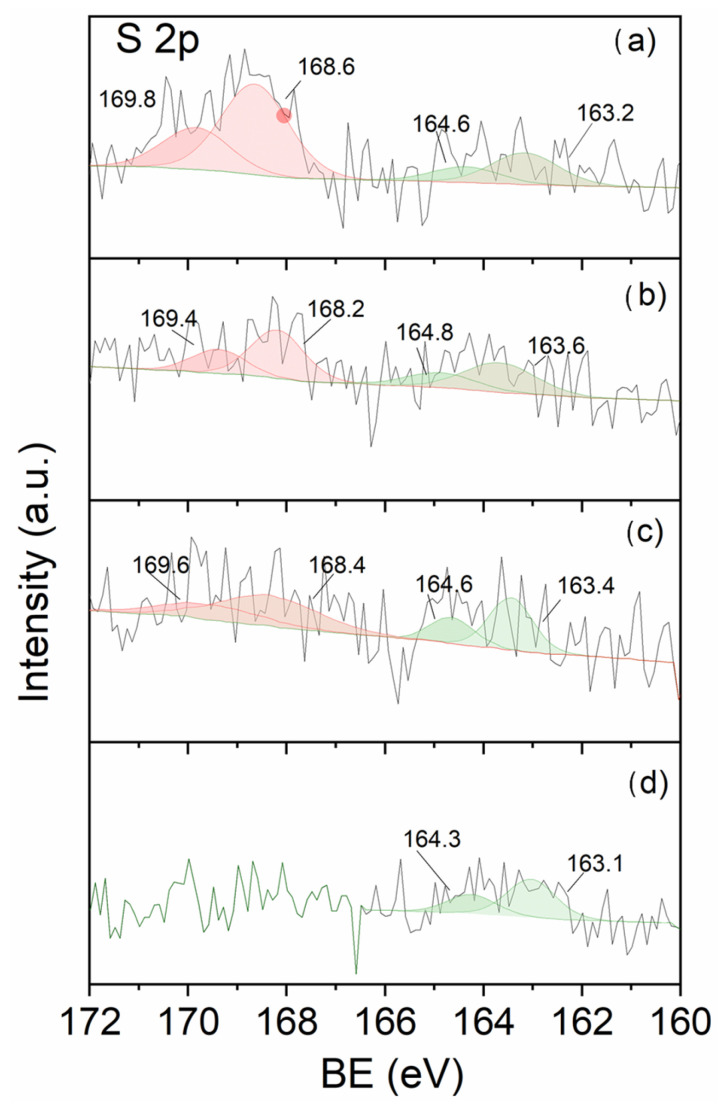
XPS S2p spectra of catalysts prepared from sulfate precursor: (**a**) PdS/HHT, (**b**) PdS/CNT, (**c**) PdS/G and (**d**) PdS/AC.

XPS was employed to determine the surface atomic composition of the catalysts, with particular emphasis on those prepared from the sulfate precursor. In these materials, special attention was paid to the sulfur content and its chemical state, distinguishing between sulfate and sulfide species. Figure 4 and Figure S2 show the core-level spectra of the S2p and Pd 3d_5_/_2_ regions, respectively. The BE of each species along with the surface atomic ratios of Pd/C, S/C and Pd/S^2−^ are collected in Table 2.

The S2p spectra reveal significant differences in sulfur speciation depending on the support (Figure 4). The characteristic S2p_3/2_–S2p_1/2_ spin–orbit doublet, with a separation of approximately 1.2 eV, appears at binding energies of 168–169 eV for sulfate species [44,45,46], and between 162 and 164 eV [47,48,49] for sulfide species. As observed, sulfate species and sulfide species coexist in PdS/HHT, PdS/CNT and PdS/G, whereas PdS/AC exclusively contains sulfide species. PdS/HHT exhibits the highest sulfur surface concentration, predominantly in the sulfate form (Table 2). Note that the catalysts used are the samples after they have been used in the reaction.

The Pd 3d_5_/_2_ spectra of all catalysts (Figure S2) show a single contribution at 335.4–335.7 eV, characteristic of metallic Pd^0^ [15,16], with no evidence of Pd(II) species, even in samples containing sulfate. The small variations in binding energy are within the experimental uncertainty and may reflect minor differences in Pd dispersion [15], as observed by TEM (Figure 2 and Figure 3), or metal–support interactions. The absence of Pd(II) species at 337.5–338 eV [15] indicates that the sulfate species detected in the S2p spectra are not directly coordinated to Pd and are most likely associated with the support. Although Pd/S atomic ratios close to unity (Table 2) point to a significant coexistence of palladium and sulfide at the catalyst surface, the XPS data do not provide direct evidence for the formation of PdS domains. In the case of the PdCl/HHT sample, no residual chlorine was detected by XPS. Among the investigated catalysts, PdS/HHT exhibits the highest S/C atomic ratio, indicating the largest sulfur surface concentration, as well as the highest proportion of sulfate species. These trends are consistent with the sulfur distribution inferred from EDX analysis. On this basis, it can be inferred that sulfur is partially anchored to the support while another fraction is located in the vicinity of Pd [23]. The higher sulfate content observed for PdS/HHT may contribute to its enhanced catalytic stability and selectivity toward ethylene, although the intrinsic influence of the support cannot be excluded.

As a summary of XPS results, we have been able to demonstrate that: (a) sulfur is present as sulfate (SO_4_^2−^) and sulfide species (S^2−^), and (b) palladium remains in the metallic state. Importantly, the detected sulfide species (S^2−^) cannot be associated with the carbon supports, which are chemically inert toward sulfur incorporation under the applied synthesis conditions. Therefore, the presence of S^2−^ species must be related to metallic Pd^0^. Accordingly, no palladium sulfide (PdS) phase is formed, since Pd^2+^ species were not detected. These results indicate that sulfur does not lead to the formation of stoichiometric PdS, but rather, sulfur may be incorporated as sub-stoichiometric sulfide species (S^2−^) interacting with metallic Pd without requiring oxidation of Pd to Pd^2+^.

#### 3.2.3. Raman Spectroscopy

**Figure 5:**
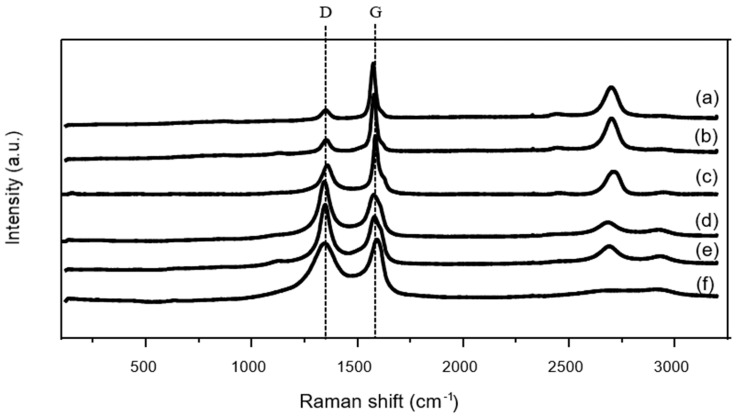
Raman average spectrum of the different carbon supported catalysts after reaction. (**a**) PdS/HHT, (**b**) PdCl/HHT, (**c**) PdS/G, (**d**) PdS/CNT, (**e**) PdCl/CNT and (**f**) PdS/AC.

Raman spectroscopy was employed to characterize the catalysts after the catalytic tests, with particular emphasis on sulfur-containing species and local analysis through Raman mapping. Figure 5 shows the average Raman spectra obtained from 80 measurement points across each sample. All the catalysts exhibit the characteristic D and G bands near 1350 and 1580 cm^−1^, respectively, associated with the carbonaceous supports [50,51,52,53,54]. Clear differences in the relative intensity of these bands are observed depending on the support. Namely, the I_D_/I_G_ ratios (Table S1) follow the order PdS/HHT ≈ PdCl/HHT < PdS/G < PdS/CNT ≈ PdCl/CNT < PdS/AC, reflecting differences in the defect density and graphitization degree; parallel to this trend, the I2D decreases continuously (Table S1). The lower D-band intensity and narrower G-band observed for the HHT support indicate a higher structural order compared to CNT and activated carbon supports [55,56]. The decrease in the intensity of the D band runs parallel to an apparent blueshift of the G band, due to the contribution of the defect D’ band near 1620 cm^−1^ [57]. As for the band in the 2900 cm^−1^ region, the trend in band intensity (HHT < graphite < CNT < AC) reflects increasing structural disorder and surface functionalization [58]. Highly graphitized, hydrogen-poor HHT shows only a very weak ~2900 cm^−1^ feature, likely from scarce C–H groups and minor defect-related D + G contributions. CNTs exhibit a stronger band due to higher surface area and defect density, while activated carbon—being the most disordered and functionally rich—displays the strongest and broadest C–H signal. Overall, greater amorphization and hydrogen content correlate with the growth of the ~2900 cm^−1^ feature.

**Figure 6:**
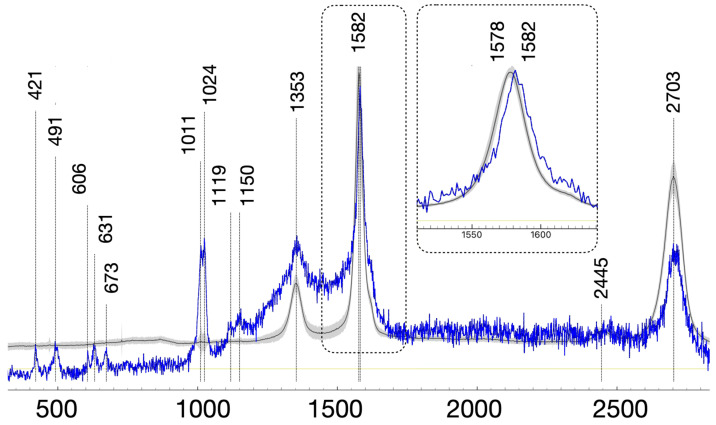
Average Raman spectrum of the reduced PdS/HHT catalyst measured at 80 spots; the gray shade is the standard deviation of the spectra. The blue trace is representative of the few spots where additional Raman bands associated with sulfur-oxide species are apparent.

In Figure 6, the black spectrum shows the average of the 80 Raman spectra taken of the PdS/HHT catalyst and its standard deviation (shaded region), which is characteristic of high-heat-treated (HHT) carbon, as described above, with a weak D band and a sharp, well-defined 2D band. Additional Raman bands are apparent at some locations of the sample, as the blue spectrum illustrates. In the blue spectrum, the Raman features below 1250 cm^−1^ can be attributed to surface sulfur–oxygen species interacting with the carbonaceous support. The weak bands in the 420–500 cm^−1^ region can be assigned to ν_2_(O–S–O) bending modes of sulfate- and sulfite-like species, which significantly broaden upon surface coordination on heterogeneous substrates like activated carbons or graphitic defects [59,60,61]. The features near 600–680 cm^−1^ may correspond to the ν_4_(O–S–O) asymmetric bending modes of sulfates on the carbon substrate [60,62]. The sharp bands near 1010 and 1024 cm^−1^ are the characteristic ν_1_(S–O) symmetric and ν_3_ asymmetric S–O stretching modes of surface sulfate species [61,63]. A weak shoulder at lower frequency (≈950–980 cm^−1^) would be consistent with the ν_1_(S–O) mode of sulfite-like species, indicating the coexistence of partially oxidized sulfur species, a situation commonly observed during SO_2_ uptake and oxidation on carbonaceous adsorbents [60]. In addition, the broad and weak intensity extending into the 1050–1160 cm^−1^ region can be assigned to the ν_3_(S–O) asymmetric stretching modes of sulfate, which are strongly broadened on carbon due to surface heterogeneity and coupling with C–O and C–SOₓ vibrations at defect sites [59,63]. The absence of sharp lattice modes characteristic of crystalline sulfates, together with the pronounced band broadening and frequency dispersion, indicates that sulfur is predominantly present as surface-bound sulfate and, possibly, some sulfite species intimately associated with the carbon framework, rather than as bulk metal sulfates or crystalline sulfur-containing phases. Furthermore, the Raman modes of the carbon support exhibit some subtle, significant changes associated with a strong interaction with sulfur oxide phases: the 2D 2703 cm^−1^ Raman band weakens and the G band blueshifts to 1582 cm^−1^. The moderate blueshift of the G band (insert in Figure 6) and weakening of the 2D band near 2700 cm^−1^ are characteristic of the interaction between sulfur oxide species and carbon [57]. The G mode blueshift reflects a disruption in the π-domains and the increase in sp3 domains at the interface [64], which shows in the D’ band that apparently blueshifts the G mode; it also decreases the intensity of the 2D mode [57].

Although none of the applied characterization techniques provides direct structural evidence for the formation of bulk Pd_4_S phases, the combined XPS, Raman and EDX results support the stabilization of Pd–S surface ensembles consistent with Pd_4_S-like active sites. XPS analysis shows that palladium remains predominantly in the metallic state after reaction, while sulfur is present in both sulfide and sulfate forms, with their relative abundance strongly influenced by the nature of the carbon support. The Pd/S atomic ratios close to unity suggest a significant coexistence of palladium and sulfur at the catalyst surface, compatible with the formation of Pd_4_S-related surface motifs rather than extended sulfide phases. Raman spectroscopy reveals clear differences in the structural order of the carbon supports and, in the case of PdS/HHT, local features consistent with sulfate species and Pd–S interactions, which are absent in CNT- and AC-supported catalysts. EDX analysis further confirms a higher overall sulfur content in the HHT-supported material. Taken together, these results indicate that the carbon support plays a key role in stabilizing Pd–S ensembles with a local environment comparable to that reported for Pd_4_S active phases in selective acetylene hydrogenation. Such stabilization provides a plausible explanation for the enhanced ethylene selectivity and stability observed for PdS/HHT, while avoiding the formation of extended palladium sulfide phases that would be detrimental to catalytic performance.

## 4. Conclusions

This study demonstrates that the selective hydrogenation of acetylene to ethylene is strongly influenced by the presence and stabilization of sulfur-modified palladium sites generated from sulfate precursors on carbon supports. Catalysts derived from palladium sulfate exhibit higher ethylene selectivity than their chloride-derived counterparts, highlighting the essential role of sulfur, beyond palladium dispersion alone, in suppressing over-hydrogenation to ethane.

The nature of the carbon support plays a decisive role in controlling the stability and surface distribution of sulfur species. Among the investigated materials, HHT fibers lead to the best-performing catalyst (PdS/HHT), displaying high and stable ethylene selectivity under reaction conditions. This superior catalytic behavior correlates with the highest sulfur retention, as consistently evidenced by EDX, XPS and Raman spectroscopy. In particular, HHT-supported catalysts retain both sulfate and sulfide species to a greater extent than CNT, graphite or activated carbon supports.

The combined characterization results indicate that the highly graphitized, low-defect surface of HHT fibers hinders complete sulfate decomposition during reduction, favoring the stabilization of sub-stoichiometric Pd–S surface ensembles consistent with the presence of metallic Pd. In contrast, supports with higher defect densities or oxygen-containing functional groups promote deeper sulfur removal, leading to a progressive loss of sulfur-modified Pd sites, decreased ethylene selectivity and increased ethane formation.

Overall, this work highlights that the controlled generation and stabilization of sub-stoichiometric Pd–S related surface motifs, governed by the choice of carbon support, constitute an effective strategy to enhance both selectivity and durability in acetylene hydrogenation. These findings are summarized in Table S2. The PdS/HHT catalyst emerges as a promising low-temperature, highly selective system, competitive with or superior to state-of-the-art Pd-based catalysts reported in the literature.

## Figures and Tables

**Table 1:** Catalytic activity, product selectivity and carbon balance for Pd-based catalysts evaluated at 40 °C and 85 min time-on-stream.

Catalyst	Activity (μmol/g_cat_∙s)	Ethane Selectivity (%)	Ethylene Selectivity (%)	C4s Selectivity (%)	Carbon Balance (%)
PdS/HHT	6.9	14	70	16	92
PdCl/HHT	7.8	23	61	16	97
PdS/CNT	11.5	19–31 *	67–55 *	14	93
PdCl/CNT	10.4	48	43	9	96
PdS/AC	20.3	13–21 *	74–66 *	13	92
PdS/G	20.6	24–32 *	75–56 *	12	90

* These results are not stable and vary throughout the reaction (as shown in Figure 1).

**Table 2:** Surface chemical composition and sulfur speciation derived from XPS analysis of reduced Pd-based catalysts after reaction.

Catalyst	S2p_3/2_ BE (eV) S^2−^/S^6+^	S Species Distribution (%) S^2−^/S^6+^	Pd 3d_5/2_ BE (eV)	Pd/C Atomic Ratio	S/C Atomic Ratio	Pd/S^2−^ Atomic Ratio
PdCl/HHT	-/-	-/-	335.5	0.0012	-	-
PdS/HHT	163.2/168.6	27/73	335.5	0.0002	0.0008	0.9
PdS/CNT	163.6/168.2	47/53	335.6	0.0003	0.0004	1.6
PdS/G	163.4/168.4	43/57	335.6	0.0002	0.0004	1.2
PdS/AC	163.1/-	100/-	335.4	0.0003	0.0003	1.0

## Data Availability

Data will be made available on request.

## Supplementary Materials

The following supporting information can be downloaded at: https://www.mdpi.com/article/10.3390/nano16030157/s1. Figure S1. EDX spectra of the post-reaction catalysts. Figure S2. XPS Pd 3d5/2 spectra of the prepared catalysts: (a) PdCl/HHT, (b) PdS/HHT, (c) PdS/CNT, (d) PdS/G and (e) PdS/AC. Table S1. Relative intensity ratios of D and G bands (ID/IG) for the different catalysts. Table S2. Correlation between the structural properties of the carbon supports, the experimental evidence for stabilization of sub-stoichiometric Pd–S surface ensembles, and the resulting catalytic behavior.
