# Supplementary material for: Selective Acetylene Hydrogenation: Influence of Carbon Supports on the Stabilization of Pd4S-like Active Sites
Source: Nanomaterials (Basel). 2026 Jan 23;16(3):157. doi: 10.3390/nano16030157 (PMC12899367; doi:10.3390/nano16030157)
Supplement: Supplementary file 1 [file nanomaterials-16-00157-s001.zip › nanomaterials-4096791-supplementary.pdf]

Selective Acetylene Hydrogenation: Influence of Carbon Supports on the Stabilization of Pd_4S-like Active Sites

Eduardo Campos-Castellanos [1], Inmaculada Rodríguez-Ramos [2], Miguel A. Bañares [2], Antonio Guerrero-Ruiz [1,*] and María V. Morales [1,*]

[1] Departamento de Química Inorgánica y Química Técnica, Facultad de Ciencias, UNED, Av. De Esparta s/n 28232 las Rozas, 28232 Madrid, Spain; camposeduardo@ccia.uned.es

[2] Instituto de Catálisis y Petroleoquímica, CSIC, C/Marie Curie 2, Cantoblanco, 28049 Madrid, Spain; irodriguez@icp.csic.es (I.R.-R.); miguel.banares@csic.es (M.A.B.)

* Correspondence: aguerrero@ccia.uned.es (A.G.-R.); mvmorales@ccia.uned.es (M.V.M.)

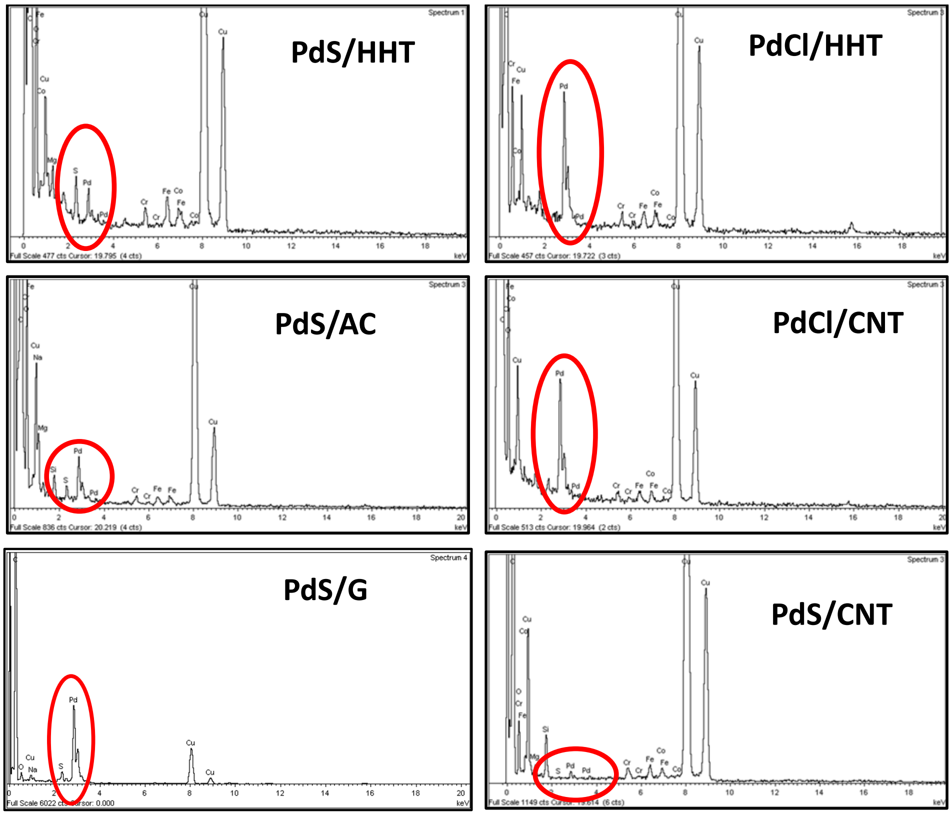

Figure S1. EDX spectra of the post-reaction catalysts.

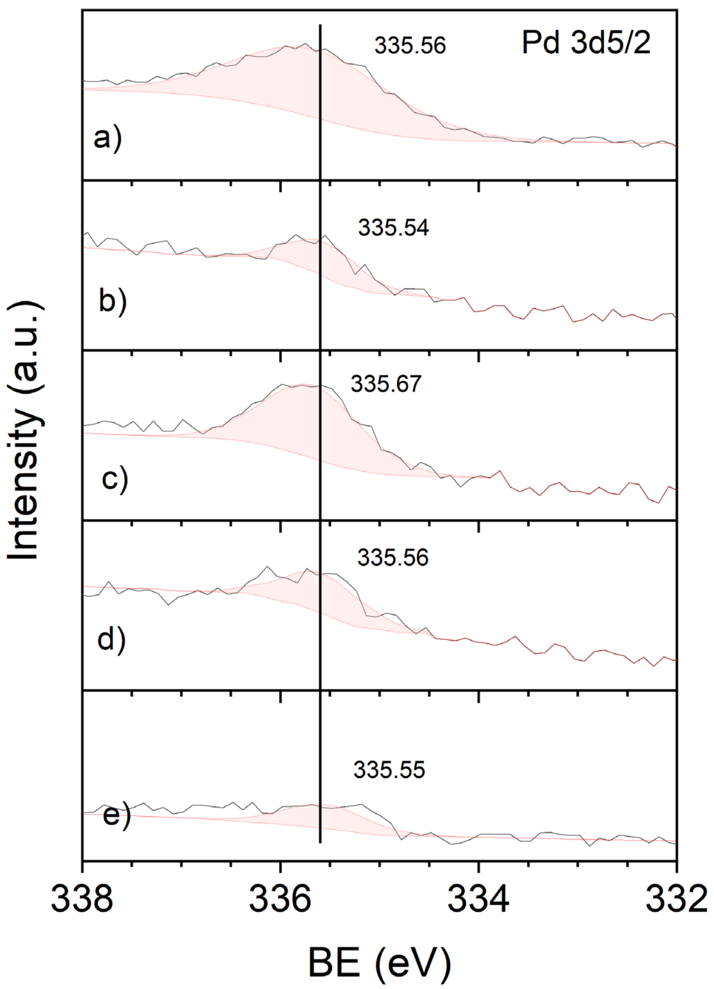

Figure. S2. XPS Pd $3d_{5/2}$ spectra of the prepared catalysts: a) PdCl/HHT b) PdS/HHT, c) PdS/CNT, d) PdS/G and e) PdS/AC.

Table S1. Relative intensity ratios of D and G bands (I_D/I_G) for the different catalysts.

Catalyst	I_D/I_G	I_{2D}/I_G
PdS/HHT	0.28	0.53
PdCl/HHT	0.32	0.54
PdS/G	0.95	0.39
PdS/CNT	1.32	0.30
PdCl/CNT	1.23	0.31
PdS/AC	2.08	0.13

Table S2. Correlation between the structural properties of the carbon supports, the experimental evidence for stabilization of sub-stoichiometric Pd–S surface ensembles, and the resulting catalytic behavior.

**Support**	**Key structural features**	**Sulfur retention / stabilization**	**Evidence for Pd–S surface ensembles**	**Catalytic activity**	**Selectivity & stability trend**
HHT	Highly graphitized, low defect density, low surface area	High sulfur retention; strong stabilization of Pd–S ensembles	Strong (XPS highest sulfur concentration, Raman SO_x + Pd–S features, EDX S-rich)	Moderate	High ethylene selectivity and excellent stability
CNT	Graphitic with curvature and defects	Intermediate sulfur retention	Moderate (XPS + limited EDX S)	Moderate –high	Initial ethylene selectivity, decreasing with time
G	Highly graphitic, planar, moderate surface area	Limited sulfur stabilization	Weak–moderate (XPS, low S by EDX)	High	Decreasing ethylene selectivity; increasing ethane
AC	High surface area, microporous, oxygenated groups	Low sulfur retention	Weak (XPS sulfide only)	High	Poor selectivity stability; increased overhydrogenation
